# Enteral Nutrition in Critical Care

**DOI:** 10.4021/jocmr1210w

**Published:** 2013-01-11

**Authors:** Carlos Seron-Arbeloa, Monica Zamora-Elson, Lorenzo Labarta-Monzon, Tomas Mallor-Bonet

**Affiliations:** a Intensive Care Unit, San Jorge Hospital, Avda. Martinez de Velasco 35. 22004 Huesca, Spain

**Keywords:** Enteral nutrition, Critical care, Nutritional support, Intensive care, Enteral feeding, Critical ill

## Abstract

There is a consensus that nutritional support, which must be provided to patients in intensive care, influences their clinical outcome. Malnutrition is associated in critically ill patients with impaired immune function and impaired ventilator drive, leading to prolonged ventilator dependence and increased infectious morbidity and mortality. Enteral nutrition is an active therapy that attenuates the metabolic response of the organism to stress and favorably modulates the immune system. It is less expensive than parenteral nutrition and is preferred in most cases because of less severe complications and better patient outcomes, including infections, and hospital cost and length of stay. The aim of this work was to perform a review of the use of enteral nutrition in critically ill patients.

## Introduction

Several ancient physicians, such as Hippocrates, Celsius, and Avicenna, among others, already prescribed certain foods for the treatment of diseases and for the patient’s convalescence. However, the concept of nutrition did not appear in the literature until the second half of the 19th century, under the term ‘Dietetics’.

It was not until the first half of the 20th century that physicians began to show interest in feeding patients incapable of eating enough, either because they should not or could not, in order to address their increased metabolic needs during severe and prolonged diseases.

The first attempts were carried out at the end of the 19th century. In 1872, Clouston described a method for intragastric feeding, infusing milk, eggs, jelly, alcohol and sugar, and in 1882, Bliss attempted providing food through the rectum. At the beginning of the 20th century, the techniques for gastrointestinal tract access began to improve, and around the 1950s, more refined mixtures started to be used, resulting in major advances, such as the development of foods for astronauts, and that of elementary diets.

In 1937, Elman carried out the first successful intravenous infusion of hydrolyzed casein to a patient. From that moment on, two schools of thought appeared: one in Sweden, which succeeded in intravenously administering lipids, together with glucose and a source of nitrogen, first as hydrolyzed casein, and then as crystalline amino acids; and another one in Philadelphia, which administered hypertonic glucose and nitrogen through a central venous catheter, using the insertion technique described by Aubaniac in 1952. In 1967, Wilmore and Dudrick reported the case of an infant that was successfully nourished intravenously for more than six weeks. From then on, this feeding method began to spread. With the booming of parenteral nutrition, enteral nutrition remained relegated until 25 years ago [1].

Over the last 30 years, enteral nutrition has developed continuously, especially because malnutrition has been established as an independent risk factor for morbidity, leading to an increasing in the rate of infections, in the length of stay in the hospital and intensive care unit, and in the number of days of mechanical ventilation, as well as a more difficult healing of wounds, and ultimately, an increase in mortality. Throughout the years, the indications have increased, the most appropriate administration routes have been established, and increasingly specific infusion systems and nutrients have been developed. Thanks to calorimetric studies, hyperalimentation could be avoided, and the supply in substrates could be better adapted to the needs specific to each situation of malnutrition and stress, thus reducing the incidence of complications and improving the outcomes.

The 1980s saw great advances in the development of chemically defined and organ-specific diets, and in the development of more advanced techniques for access. Feeding tubes have been improved so that they are thinner, more comfortable and safer. In addition, gastrointestinal tract accesses through radiological, surgical and endoscopic techniques for nasoenteric intubation and gastrojejunostomy tube placement have been improved [1].

In the last decades, multiorgan failure became the main cause of death among critically ill patients and in 1988, Wilmore [2] hypothesized that bacterial translocation could be the main source and trigger for sepsis. Therefore, research focused on studying the gastrointestinal tract, which went from being considered a mere nutrient digestion and absorption organ to the spotlight as a barrier against bacteria and intraluminal toxins and an organ with significant hormonal, metabolic and immune functions

## Justification for the Nutritional Support of the Critically Ill Patients

Critically ill patients are at particular risk of malnutrition, which occurs in up to 40% of the cases. The metabolic changes that occur in response to stress lead to an increase in protein catabolism, resulting in a significant loss of lean body mass, which in turn results in a higher incidence of complications, especially infectious ones, in an increase in wound dehiscence and in unfavorable outcomes. The main purpose of nutritional support is to prevent malnutrition and its associated complications, by modulating the stress response of the patients [3]. This objective will be achieved by: (1) providing the appropriate doses of macro- and micronutrients to meet the calculated or measured needs; (2) avoiding complications associated with nutritional support; (3) reducing nitrogen deficits; and (4) modulating the inflammatory response through the use of different substrates.

## Indications, Contraindications and Complications of Enteral Nutrition in Critically Ill Patients

In general, intensive care unit patients who present with malnutrition or a high probability of developing malnutrition during their hospital stay and those who are not expected to be on a full oral diet within three days should receive specialized enteral and/or parenteral nutritional support. In case of enteral nutrition, feeding should be started early within the first 24 - 48 hours following admission to facilitate diet tolerance, reduce the risk of intestinal barrier dysfunction and infections, and reduce the length of hospital stay and mechanical ventilation [4].

The most widely used guidelines of different scientific societies on the use of enteral nutrition in critically ill patients [5-8] and their level of evidence according to the GRADE Working Group [9] are summarized in Table 1. Moreover, the most common contraindications and complications associated with enteral nutrition are reported in Tables 2, 3, respectively [10-14].

**Table 1:** Summary of Recommendations for Enteral Nutrition in Critically Ill Patients

Summary of recommendations for enteral nutrition in critically ill patients	Level of evidence
1. Enteral nutrition is associated with an improvement of nutritional variables, a lower incidence of infections and a reduced length of hospital stay.	A
2. Critically ill patients who cannot be fed orally for a period of more than three days must receive specialized nutritional support.	C
3. Enteral nutrition is preferable to parenteral nutrition.	B
4. Enteral nutrition should be started within the first 24 - 48 hours of admission.	A
5. Enteral nutrition should provide 25 to 30 kcal/kg/day.	C
6. The feedings should be advanced toward goal over the next 48 - 72 hours.	C
7. The enteral nutrition must be deferred until the patient is hemodynamically stable.	C
8. In intensive care unit patients, neither the presence nor absence of bowel sounds and evidence of passage of flatus and stool is required for initiation of enteral nutrition.	B

**Table 2:** Contraindications to Enteral Nutrition

**Absolute contraindications to enteral nutrition:**

1. Diseases associated with ileus: multiple trauma with significant retroperitoneal hematoma and peritonitis
2. Intestinal obstruction
3. Active gastrointestinal hemorrhage
4. Hemodynamic instability: enteral nutrition in an ischemic small bowel can worsen the ischemia and lead to necrosis and bacterial overgrowth

**Relative contraindications, use of a mixed nutritional support:**

1. Diverticular abscess
2. Early stages of Short bowel syndrome
3. Severe malabsorption
4. Small bowel fistulas, depending on the flow rate and localization
5. Need for early nutritional support and full enteral feeding impossible:
Severely malnourished patients with severe hypercatabolism
Patients in whom an appropriate intestinal approach cannot be carried out or who do not tolerate the full requirements

**Table 3:** Complications of Enteral Nutrition

**Mechanic**

1. Erosion and/or necrosis and/or infection at the contact zones
2. Pharyngeal, esophageal and/or tracheobronchial perforation and stenosis
3. Tracheoesophageal fistula
4. Malpositioning and removal of the probe
5. Obstruction and tethering of the probe
6. Intraperitoneal leakage through osteotomy site
7. Leakage of the formulation
8. Pulmonary aspiration
9. Hemorrhage

**Metabolic**

1. Hypertonic dehydration
2. Hyperosmolarity
3. Nonketotic hyperosmolar coma
4. Hyper/hypoglycemia
5. Dyselectrolytemia
6. Hyperhydration
7. Dumping syndrome
8. Refeeding syndrome
9. Hypercapnia

**Infectious**

1. Sinusitis and otitis
2. Aspiration pneumonia
3. Necrotizing peritonitis and enteritis
4. Dietary contamination

**Gastrointestinal**

1. Increased gastric residual volume
2. Constipation
3. Abdominal fullness and distention
4. Vomiting and regurgitation
5. Diarrhea
6. Hypertransaminasemia, hepatomegaly

## Considerations About the Provision of Nutritional Support to Critically Ill Patients

Usually, the caloric intake of critically ill patients receiving artificial nutrition is much lower than desired, recommended or measured, especially with the enteral route [15-19]. Some studies have found a relationship between hypocaloric intake and mortality, infection and nosocomial bacteremia [11]. Dvir et al [20] revealed in a study on patients undergoing mechanical ventilation that the cumulative caloric deficit strongly correlated with the occurrence of complications, but not with mortality, the length of hospital stay or the length of mechanical ventilation. Rubinson et al [21] found an association between a caloric intake below 25% of the recommended and the incidence of bacteremia, and Krishnan [11] found that moderate caloric intake (between 33 and 66% of the recommended) was associated with better clinical outcomes. These data suggest that the optimal amount of calories required by critically ill patients continues to be controversial [21, 22].

The most reliable method for calculating energy consumption is indirect calorimetry. If not available, an amount of approximately 25 kcal/kg of current weight/day is recommended in patients with a body mass index below 30. In mechanically ventilated patients, the caloric needs should be estimated with the Penn State equation [23]. Carbohydrate intake should not exceed 4 g/kg/day and blood glucose levels should remain below 180 mg/dL. The recommended lipid supply is 0.7 - 1.5 g/kg/day, and the use of lipid emulsions with a high omega-6 fatty acid content should be avoided in critically ill patients. The supply in amino acids must be adjusted to 1 - 1.8 g/kg/day, depending on the level of metabolic stress. The supply of micronutrients, such as vitamins and trace elements, is also recommended, although the amounts required cannot be determined [24].

Regarding some specific amino acids, there is scientific evidence that supports a supply of parenterally administered glutamine of 0.5 g/kg/day. However, there are not enough studies to support its enteral administration, which does not seem to be associated with an increase in the corresponding plasma levels [25]. Another amino acid considered to be conditionally essential in critically ill patients is arginine. Its administration is recommended to critical trauma and surgical patients; however, it is currently under discussion for patients with severe sepsis [7].

A hypocaloric intake during the first phases of stress could have beneficial effects, such as a better glycemic control, that would reduce the occurrence of infectious complications, although this mechanism remains to be proven [26]. Some authors recommend the supply of 80% of the nutritional needs during the first seven to ten days, and their increase during convalescence [27, 28].

Among the reasons for this low initial caloric supply by enteral nutrition, the increase in gastric residual volume, as well as the different nursing, diagnostic and surgical procedures carried out on critically ill patients, are particularly noteworthy [29]. Some authors, such as Montejo [30], showed that an upper limit of tolerability for gastric residual volume, as an indicator for enteral nutrition intolerance, improves the volume provided through enteral nutrition. The use of postpyloric access routes, the use of procedural protocols and an early start of nutritional support have been shown to improve the enteral nutrient supply [6, 29].

Another important consideration to take into account for the nutritional support of critically ill patients is the delay in the onset of nutritional support. The clinical practice guidelines recommend that nutritional support be started early in critically ill patients [5, 6, 8] which is in practice achieved for approximately 50% of the patients, because the initial hemodynamic alterations which characterize critically ill patients, impede early feeding in many cases [10, 13, 31, 32]. Early, as opposed to late, enteral nutrition has been shown to have beneficial effects on patient outcome, in terms of length of mechanical ventilation, incidence of infections and/or mortality [33]. Moreover, nutrition seems to be beneficial regardless of the access route [8]. In our experience, early nutritional support is associated with lower mortality, although we did not observe a reduction in the incidence of infectious complications [34].

## Benefits of Enteral Nutrition

In addition to its digestive, absorptive, endocrine and metabolic functions, the intestine is also an effective barrier against bacteria and intraluminal toxins, thanks to the high turnover rates of the enterocytes of the intestinal epithelium, the mucus secreted by goblet cells, and the large amount of lymphoid tissue that forms an immune barrier. Eighty percent of immunoglobulins synthesized in the organism, especially IgA, are secreted through the gastrointestinal tract, and 50% of the immune mass is found in this organ [2].

Intestinal dysfunction is common in critically ill patients, but there is no objective definition for it. Enteral nutrition intolerance is the most simple and useful sign to evaluate it. Its causes are multifactorial and have been identified through different experimental studies, which showed that intestinal bacteria are the cause of infectious complications in hospitalized patients, and that the increase in intestinal permeability could favor bacterial translocation. Intestinal ischemia resulting from shock and sepsis states can produce hypoxia and reperfusion injuries that affect the intestinal wall permeability, through oxygen-free radicals, cytokines, acidosis, ATP depletion and neutrophil activation. Moreover, fasting also causes the disruption of intestinal integrity, through atrophy and a decrease in the size of microvilli, of the depth of the crypts, of the intestinal weight and cellular mass, resulting in a decrease in the number of cellular mitoses [35].

Enteral nutritional support has been shown to stimulate intestinal growth and function, both directly intraluminally, because it supplies substrates for enterocyte oxidation, and indirectly, because it promotes hormone secretion through the intestinal trophic effect, which would reduce bacterial translocation and the problems associated with it. Enteral nutrition seems to present benefits in comparison with parenteral nutrition, such as a lower number of infectious complications, non-infectious complications and associated costs [5, 36-41].

Gramlich et al performed a systematic review of the literature and found that enteral nutrition was associated with a lower number of infections, although there was no difference in terms of mortality, length of hospital stay or length of mechanical ventilation [38]. Elke et al showed that parenteral nutrition was independently associated with mortality in septic patients [39]. However, other authors reported different results. Simpson et al performed a meta-analysis, and showed that enteral nutrition was associated with a lower mortality than parenteral nutrition, but with a higher number of infectious complications [3]. Peter et al also found, through another meta-analysis, that there was no difference in mortality between early enteral and early parenteral nutrition, although the incidence of complications, both infectious and non-infectious, was higher in patients under parenteral nutrition [40].

This lack of uniformity regarding the benefits of enteral over parenteral nutrition suggests that once the need of nutritional support has been established, enteral nutrition should be preferably used. However, if enteral nutrition cannot be used, parenteral nutrition should be immediately started.

## Enteral Nutrition in Special Disease States

### Renal failure

Acute renal failure is increasingly common in critically ill patients. Nutritional support is aimed at preserving the lean mass and energy reserves, avoiding malnutrition, attenuating the inflammatory response and oxidative stress, and improving endothelial function [42, 43].

Normal diets are inadequate for non-hypercatabolic patients with renal failure conservatively treated or in intermittent hemodialysis because of oligoanuria, because of their low density and excessive sodium, potassium and phosphate content. In these patients, hypoproteic or normoproteic diets are recommended, with high biological value proteins, high energy density and low potassium, sodium and phosphate content [44, 45].

However, hyperproteic diets (2 - 2.5 g/kg/day) must be provided to hypercatabolic patients on daily dialysis or continuous renal replacement procedures, adjusted to the underlying pathology and supplemented with glutamine. In some cases, the content in tyrosine, taurine, histidine and branched-chain amino acids should be increased [46].

Monitoring of serum electrolytes (phosphorus, potassium and magnesium) and the micronutrient levels (zinc, selenium, thiamin, folinic acid and vitamins A, C, and D) is recommended, to individualize the supply.

### Liver failure and transplantation

Malnutrition is a frequent finding in patients with liver failure and significantly impacts on mortality, especially in patients with alcoholic cirrhosis, as opposed to viral cirrhosis [47]. Thus, in patients who are candidates for liver transplant, malnutrition negatively affects the outcome of the procedure [48].

Enteral nutrition should be considered first, if nutritional support is required. Esophageal or gastric varices and coagulopathy are typical contraindications in clinical practice for nasogastric tube insertion, although this contraindication is not based on clinical studies and has been discussed by some authors. Parenteral nutrition should be provided if the gastrointestinal tract is not functioning properly because of a digestive hemorrhage, if enteral nutrition is not well tolerated, if enteral nutrition is not enough to meet the nutritional needs, or if there is a high risk of aspiration, as a result of alterations in the level of consciousness associated with advanced stages of encephalopathy [8].

In these patients, a caloric intake of 25 to 40 kcal/kg/day is recommended, with a mixed energetic supply (carbohydrates and fats). In patients with liver failure, the regular use of diets enriched in branched-chain amino acids is not recommended. These should be restricting to patients with encephalopathy arising during enteral nutrition. However, the supply of vitamins and trace elements should be increased, especially that of zinc, magnesium and potassium.

In patients with liver transplantation, nutritional support should be started early after the transplantation procedure, preferably via the enteral route and through transpyloric access [49].

### Acute severe pancreatitis

Severe acute pancreatitis provokes a systemic inflammatory response that leads to a highly catabolic, hypermetabolic and hyperdynamic stress states [50].

The classical treatment of this syndrome consists of bowel rest and parenteral nutrition, but in the last decade, numerous studies have shown that this approach is associated with a high mortality and morbidity [51].

Intestinal barrier dysfunction occurs during the early phase of acute pancreatitis, and is associated with infectious pancreatic necrosis, multiorgan failure and mortality [52]. For these reasons, the preferred route of the nutritional support is enteral feeding into the jejunum, which should be started early, within the first 48 h. Even in patients who do not tolerate enteral nutrition well, it is recommended to maintain a minimum enteral nutrient supply [51].

### Respiratory failure

Respiratory failure requiring mechanical ventilation is one of the most common reasons for intensive care unit admission, in addition to flare-ups chronic obstructive lung disease and acute respiratory distress. These patients are at high risk of malnutrition because of their underlying disease, their catabolic situation and the mechanical ventilation itself.

In patients with acute exacerbation of chronic respiratory failure, the recommended level of protein supply ranges from 1.0 to 1.8 g/kg/day and the use of specific high-fat, low-carbohydrate formulas is not indicated. Special attention should be paid to the supply of potassium, phosphorus, magnesium and antioxidants. In patients with acute lung injury and acute respiratory distress syndrome, an enteral diet enriched in omega-3 fatty acids and antioxidants is recommended [53].

### Abdominal surgery

The nutritional needs of patients undergoing abdominal surgery are similar to that of other critically ill patients, although it should be taken into account that the surgery itself can trigger both inflammatory and metabolic changes. Malnutrition is associated with changes in body composition, as well as a delay in wound healing, a decrease in functional ability and a deterioration of the immune function; therefore, these patients have a higher risk of infectious and cardiorespiratory complications, which can result in an increase in hospital length of stay and in a higher mortality [54].

Early postoperative enteral feeding is effective and well tolerated, even in the presence of ileus and if the integrity of the newly constructed anastomosis is compromised, and it is associated with a reduction in the incidence of postoperative infectious complications and improved tissue healing [55]. In patients undergoing gastrointestinal tract surgery with proximal anastomosis, enteral nutrition using a feeding catheter placed distally to the anastomosis is recommended [54]. In case of enteral nutrition intolerance, the administration of prokinetic drugs should be considered. A complementary parenteral nutrition should be started when less than 60% of the nutritional needs are met on the third day after admission or during the hospital stay for at last two consecutive days [56].

In case of parenteral nutritional support, the supply of omega-3 fatty acids [57] and supplementation in glutamine has been recommended, although there are not enough data to justify their use in surgical patients receiving enteral nutrition [54].

### Multiple trauma

Multiple trauma patients are previously healthy patients who suddenly suffer a severe aggression; therefore, nutritional support should be started early, preferably enterally and with a protein supply adapted to the catabolism of the patient and supplemented in glutamine. In non-obese patients, a total daily caloric supply of 25 to 30 kcal/kg/day is recommended, and in patients with a spinal cord injury, a supply of 20 to 24 kcal/kg/day is recommended [58]. The latter show a specific evolution; it is thought that after a period of metabolic lethargy, a phase of intense proteolysis begins, which is difficult through nutritional support, since its pathophysiological base is more related to denervation than to the neuroendocrine storm of acute critically ill patients. During the first four weeks following spinal cord injury, weight loss occurs, which can be estimated at 10-20% of body weight, and about 85% of it corresponds to lean mass.

The supply of glutamine and other pharmacological nutrition agents, such as omega-3 fatty acids, arginine and antioxidants, is also recommended in multiple trauma patients [58-60].

### Sepsis

Specialized nutritional support should be delayed in patients in septic shock and hemodynamic instability, until correct resuscitation and hemodynamic stability has been achieved.

Enteral feeding is the first choice of nutritional support in a septic patient, and it can be supplemented with different substrate mixtures, such as arginine, since it does not affect the evolution of the patient [61], although only the benefits of omega-3 supplementation have been demonstrated [62].

## Will it be Possible to Eliminate Parenteral Nutrition Support in Critically Ill Patients?

Considering the historical evolution of nutritional support, its development has been fundamentally based on three elements [31]: 1) The development of practical, effective and safe access systems for the administration of nutrients through both routes. There have been great advances in enteral access to the gastrointestinal tract: in addition to the classical nasogastric tube, nasojejunal access and gastrostomy and jejunostomy tubes inserted through surgical, endoscopic and radiological techniques have been developed; 2) The increase in nutritional support indications and the scientific development of our knowledge. The proof that patients with diseases such as renal and hepatic failure, who were typically treated with parenteral nutrition, could also be treated with enteral nutrition in a safe manner, the favorable effect of enteral nutrition on the flare-ups of intestinal inflammatory disease, the possibility to use an enteral access in different types of fistulas, the proof of enteral nutrition tolerance in the immediate postoperative period, and the change in mentalities on the mere supply of substrates to treat or recover a state of malnutrition, which is currently referred to as nutritional support, with the aim of modulating the inflammatory response to the aggression; and 3) The development of increasingly sophisticated nutrient solutions, both for parenteral and enteral nutrition, that can administrate a number of nutrients with specific properties, such as glutamine, arginine, monounsaturated fatty acids, fish oils, taurine, nucleosides and nucleotides, as well as a wide selection of fats, micronutrients and antioxidants.

In the future, the research in this field will be focused on, among others: 1) Establishing the real benefits of the different nutrients in different types of diseases and in the different types of stress that affect critically ill patients; 2) Establishing the real benefits of the different elements of immunomodulating foods; 3) Establishing which is the caloric and nitrogen supply needed during the various stages of the stress response (early vs. late), now that the benefits of early enteral nutrition seem to be clearly established; 4) Determining if the initial hyperalimentation of critically ill patients provides real benefits; and 5) Determining if in case of difficulty meeting the nutritional needs with enteral feeding during the first phases, a complementary parenteral nutrition should (or not) be started.

Recent years have seen a steady growth and development of systems to use the best option in terms of route of access, supply and type of nutrients for nutritional support. Currently, enteral feeding is the method of choice for the nutritional support of critically ill patients, and it can be supplemented with parenteral nutrition if the nutritional needs cannot be completely met, leaving parenteral nutrition for very specific cases or for cases where an effective access is impossible. Therefore, only the scientific and technological limitations on the previously mentioned elements will determine the achievement of the objective of solving the current limitations and complications of nutritional support.
